# Roles of glomerular endothelial hyaluronan in the development of proteinuria

**DOI:** 10.14814/phy2.15019

**Published:** 2021-09-02

**Authors:** Akimasa Asai, Naoyuki Hatayama, Keisuke Kamiya, Mai Yamauchi, Hiroshi Kinashi, Makoto Yamaguchi, Takayuki Katsuno, Hironobu Nobata, Kazushi Watanabe, Akihiko Wakatsuki, Jan Aten, Shoichi Maruyama, Takuji Ishimoto, Shuichi Hirai, Munekazu Naito, Yasuhiko Ito

**Affiliations:** ^1^ Department of Nephrology and Rheumatology Aichi Medical University Nagakute Aichi Japan; ^2^ Department of Anatomy Aichi Medical University School of Medicine Nagakute Aichi Japan; ^3^ Department of Obstetrics and Gynecology Aichi Medical University Nagakute Aichi Japan; ^4^ Department of Pathology Amsterdam University Medical Center (Location AMC) University of Amsterdam Amsterdam The Netherlands; ^5^ Department of Nephrology Nagoya University Graduate School of Medicine Nagoya Aichi Japan

**Keywords:** anti‐VEGF therapy, glycocalyx, hyaluronan, isolated perfused kidney, proteinuria

## Abstract

Vascular endothelial cells are covered with glycocalyx comprising heparan sulfate, hyaluronan, chondroitin sulfate, and associated proteins. Glomerular endothelial glycocalyx is involved in protecting against induction of proteinuria and structural damage, but the specific components in glycocalyx that represent therapeutic targets remain unclear. Anti‐vascular endothelial growth factor (VEGF) therapy is associated with an increased risk of glomerular endothelial injury. This study investigated whether hyaluronan could provide a therapeutic target to protect against proteinuria. We conducted ex vivo and in vivo experiments to explore the effects of degrading glomerular hyaluronan by administering hyaluronidase and of supplementation with hyaluronan. We investigated hyaluronan expression using biotin‐labeled hyaluronan‐binding protein (HABP) in human kidney specimens or serum hyaluronan in endothelial injuries under inhibition of VEGF signaling. We directly demonstrated hyaluronan in glomerular endothelial layers using HABP staining. Ex vivo and in vivo experiments showed the development of proteinuria after digestion of hyaluronan in glomerular capillaries. Supplementation with hyaluronan after hyaluronidase treatment suppressed proteinuria. Mice in the in vivo study developed albuminuria after intraperitoneal injection of hyaluronidase with decreased glomerular hyaluronan and increased serum hyaluronan. In human kidneys with endothelial cell dysfunction and proteinuria due to inhibition of VEGF, glomerular expression of hyaluronan was reduced even in normal‐appearing glomeruli. Serum hyaluronan levels were elevated in patients with pre‐eclampsia with VEGF signaling inhibition. Our data suggest that hyaluronan itself plays crucial roles in preventing proteinuria and preserving the integrity of endothelial cells. Hyaluronan could provide a therapeutic target for preventing glomerular endothelial glycocalyx damage, including VEGF signaling inhibition.

## Graphical abstract

Glomerular endothelial glycocalyx is involved in protection against induction of proteinuria and structural damage; however, which components in glycocalyx represent therapeutic targets remain unclear. Our data suggest that hyaluronan itself plays a crucial role in preventing proteinuria. Hyaluronan could provide a therapeutic target for preventing glomerular endothelial glycocalyx damage, including VEGF signaling inhibition induced by anti‐VEGF–VEGFR2 drugs and pre‐eclampsia.

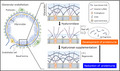

## 1 INTRODUCTION

The glomerular filtration barrier consists of three layers: podocytes; the glomerular basement membrane; and endothelial cells. Podocyte injury is now widely recognized as a major cause of proteinuria (Brinkkoetter et al., 2013; Fissell & Miner, 2018), but the roles of glomerular endothelial cells in proteinuria are not yet completely understood. Glomerular diseases with endothelial injuries such as thrombotic microangiopathy (TMA) have been increasing. One reason is the wide use of agents targeting the vascular endothelial growth factor (VEGF) signaling pathway in patients with cancer (Estrada et al., 2019; Katsuno et al., 2020). However, treatment strategy against TMA has not been fully established (Brocklebank et al., 2018). Even though these agents are effective against cancer, treatment is often discontinued once proteinuria develops. Identification of mechanisms for reducing the nephrotoxicity of VEGF‐inhibiting drugs is thus needed.

Vascular endothelial cells are covered by glycocalyx, a bioactive gel‐like layer composed of heparan sulfate, hyaluronan, chondroitin sulfate, and associated proteins (Butler et al., 2020; Cosgun et al., 2020; Dane et al., 2015; Jourde‐Chiche et al., 2019). Endothelial glycocalyx reportedly plays a role in maintaining a negative charge, regulating coagulation, and constituting a macromolecular sieve to control microvascular permeability (Henry & Duling, 1999; Jourde‐Chiche et al., 2019; Onions et al., 2019; Rabelink & De Zeeuw, 2015; Singh et al., 2007). The glomerular endothelial glycocalyx has recently been considered to play a role in protecting against the development of proteinuria and structural damage. However, the effects of hyaluronidase, as the main hyaluronan‐degrading enzyme, on proteinuria are controversial and those components of glycocalyx that represent therapeutic targets remain unclear (Dane et al., 2013; Desideri et al., 2018; Jeansson & Haraldsson, 2003). In addition, supplementation with hyaluronan and glycosaminoglycan was reportedly required to reconstitute hyaluronan after hyaluronidase treatment, while hyaluronan alone was insufficient (Henry & Duling, 1999; Potter & Damiano, 2008). Those results were judged by estimating the thickness of glycocalyx using methods with different sizes of dextran or microparticle (Desideri et al., 2018; Henry & Duling, 1999; Jeansson & Haraldsson, 2003; Potter & Damiano, 2008; Vink & Duling, 1996). One reason why results in glycocalyx have been controversial has been the difficulty of visualizing glomerular hyaluronan. In addition, degradation of hyaluronan after enzyme treatment was not evaluated in previous studies (Dane et al., 2013; Desideri et al., 2018; Henry & Duling, 1999; Jeansson & Haraldsson, 2003, 2006).

The present study investigated the roles of glomerular hyaluronan in the development of proteinuria using techniques for in situ visualization of glomerular hyaluronan and measurement of degraded hyaluronan. To exclude the influence of inflammatory cells, mediators, and metabolic influences, we conducted both ex vivo and in vivo experiments. Finally, we investigated the expression of hyaluronan in human kidney diseases under VEGF signaling inhibition, including the TMA due to anti‐VEGF therapy and pre‐eclampsia.

## 2 MATERIALS AND METHODS

### 2.1 Animals

Male inbred Lewis rats (LEW/SsN Slc) at 10 weeks of age and 300–350 g of body weight and 8‐week‐old male C57BL/6JJmsSlc mice (Japan SLC) were used for isolated kidney perfusion experiments and in vivo experiments, respectively. Animals were housed in stainless steel cages at an animal housing facility, and fed a standard diet including water ad libitum. The Institutional Animal Care and Usage Committee of Aichi Medical University (Nagakute, Japan) approved the protocol for these animal investigations (approval numbers 2017–85 and 2019–76).

### 2.2 Ex vivo kidney experiments with the isolated perfusion rat kidney system

Animal experimental protocols are shown in Figure S1 (all Supplemental Materials are available at https://figshare.com/s/a44f90ca4e8cf122bde9). In rats anesthetized with 0.5 mg/kg of pentobarbital, the right ureter was cannulated with a polyethylene tube (PE‐10, outer diameter 0.61 mm, inner diameter 0.28 mm; Becton, Dickinson and Company), and a 24‐gauge indwelling needle was positioned in the renal artery. Extracted right kidneys were connected to the isolated perfusion rat kidney (IPRK) system, as previously described (Chang et al., 2013; Figure S2). The renal artery was perfused at 37℃ with Krebs–Henseleit buffer containing 4% bovine serum albumin (BSA) (FUJIFILM Wako Pure Chemical Corporation), saturated with carbogen (95% O_2_, 5% CO_2_) (Chang et al., 2013). Final concentrations of perfusate were 113 mM NaCl, 4.3 mM KCl, 2.5 mM CaCl_2_, 0.8 mM MgCl_2_, 25.5 mM NaHCO_3_, 0.5 mM NaH_2_PO_4_, 5.6 mM glucose, and 0.9 mM nitroprusside (Merck). The perfusate was sterilized through a 0.45‐μm filter (Sarstedt AG & Co. KG) before perfusion. Perfusion pressure was held constant at 90–110 mmHg. The renal ureter was accessed for urine collection, and renal vein effluent was drained without return to the IPRK system (Figure S2). To account for discharging blood component and rewarming, all kidneys were pre‐perfused with IPRK for 5 min just before perfusion. To assess the effects of hyaluronidase, kidneys were administered with a bolus of bovine testis hyaluronidase (H3506; Sigma‐Aldrich) at a dose of 15 × 10^3^ U/kg for degradation of glomerular hyaluronan, and perfused samples were collected and kidneys were harvested (Figure S1a). Doses of hyaluronidase were determined as previously described (Dane et al., 2013; Jeansson & Haraldsson, 2003). After 5 min of pre‐perfusion and treatment with or without a bolus of hyaluronidase, urine and perfusate coming out of the kidney by IPRK were sampled every 10 min up to 30 min. Total urine production and perfusion rate were measured throughout perfusion. At the termination of perfusion, kidneys were sampled for histological evaluation (Figures S1b‐1, Figure S2). In addition, hyaluronan from *Streptococcus equi* (mol. wt. 70,000–120,000; Sigma‐Aldrich) was administered at a dose of 0.4 mg/ml after hyaluronidase treatment to reconstitute the glomerular hyaluronan. In addition, hyaluronan and chondroitin sulfate from shark cartilage (Sigma‐Aldrich) were administered at a dose of 0.4 mg/ml (1:1 mixture of each reagent) after hyaluronidase treatment (Figure S1b‐3, Figure S4).

### 2.3 In vivo kidney experiments in mice

Mice were intraperitoneally injected with 2 ml of saline containing hyaluronidase (Sigma‐Aldrich) (Hyaluronidase group). Control mice were injected with 2 ml of saline alone (Control). Two hours after injection, mice were killed, and blood, urine, and kidney tissues were harvested for subsequent analyses of histochemistry and electron microscopy (EM), and measurements of creatinine, albumin, and hyaluronan (Figure S1c).

### 2.4 Expression of hyaluronan in human kidney diseases

Expression of hyaluronan‐binding protein (HABP) was studied using human kidney biopsy samples obtained at Aichi Medical University Hospital (Nagakute, Japan) or Nagoya University Hospital (Nagoya, Japan) for diagnostic evaluation. Serum samples were taken from patients with pre‐eclampsia and normal pregnancy in the Department of Obstetrics and Gynecology in Aichi Medical University Hospital. Human studies were approved by the ethics committees for human research at both hospitals (approval numbers: 2017‐M045, ‐M049, 2020–017, and 2010–1135–5). All patients provided informed consent before participation in this study. Kidney biopsy samples included protocol biopsy at 2–3 months after kidney transplantation (Control, *n* = 6), immunoglobulin A nephropathy (IgAN) (*n* = 5), and drug‐induced TMA (*n* = 5). Histological diagnosis was conducted by light microscopy, immunofluorescence microscopy, and EM.

### 2.5 Histochemistry

Part of the kidney tissue was fixed in Maskedform (Japan Tanner), a commercially available formalin fixative, and embedded in paraffin using standard techniques. Four‐micrometer‐thick sections were stained with hematoxylin and eosin (HE), periodic acid–Schiff (PAS), silver methenamine, and Masson's trichrome (MT) for routine pathological evaluation. Another part of the kidney tissues was embedded in optimal cutting temperature compound (Sakura Finetek) and frozen in liquid nitrogen. The remainder was fixed with glutaraldehyde for EM, as described previously (Kinashi et al., 2013; Sun et al., 2019; Suzuki et al., 2012). Reagents for histochemistry are shown precisely in Table S1. Glomerular hyaluronan expression was visualized on paraffin‐embedded sections using biotin‐labeled recombinant human HABP (Hokudo, Sapporo, Japan). Detection of glycosyl residues of (GlcNAc)_2‐4_ in glomerular capillary walls by tomato‐derived *Lycopersicon esculentum* lectin (LEA) was conducted on paraffin sections as a marker of endothelial cells (Dane et al., 2013, 2015; Robertson et al., 2014). Immunostaining for laminin and synaptopodin was performed on paraffin‐embedded tissues. Sections were deparaffinized, rehydrated, and incubated in 3% hydrogen peroxide in methanol to block endogenous peroxide for 30 min. Antigen retrieval for laminin and synaptopodin was performed using HISTOFINE Protease (Nichirei Bioscience) for 60 min or citrate acid solution (pH 6.0, Immuno Active IA‐6500; Matsunami Glass) at 90℃ for 30 min, respectively. Sections were incubated with 10% normal goat serum (Nippon Bio‐Supply Center) in phosphate‐buffered saline for 30 min to block nonspecific binding. Sections were subsequently incubated with rabbit anti‐laminin or mouse anti‐synaptopodin antibodies followed by a conjugate of polyclonal goat anti‐mouse or anti‐rabbit IgG antibody and horseradish peroxidase‐labeled polymer as a secondary reagent. Enzyme activity was finally detected using 3,3‐diaminobenzidine (Nichirei Bioscience). For heparin sulfate staining, 4‐μm‐thick frozen sections were cut with a cryostat, air‐dried, and fixed in acetone at room temperature for 10 min. Sections were then incubated with mouse anti‐heparan sulfate (10E4) antibody for 60 min, followed by FITC‐conjugated goat‐anti mouse IgM antibody. Fluorescence imaging was observed using BX51 (Olympus) or confocal microscopy (LSM 710; Carl Zeiss), and recorded using ZEN2 Image software (Carl Zeiss). Glomerular expressions of HABP, LEA, heparan sulfate, and synaptopodin were scored in at least 10 glomeruli per tissue in animals or 4 glomeruli in human tissues by the extent of staining: 3, normal expression (positive staining in all capillary walls); 2, mild damage (≤25% loss of capillary wall); 1, moderate damage (26%–75% loss of capillary wall); or 0, severe damage (>75% loss of capillary wall). Average scores were calculated and the result was defined as the expression score. EM was conducted to assess the glomerular endothelial cell fenestration density, fenestral diameter, and podocyte foot process width in mice kidney, as reported previously (Oltean et al., 2015; Onions et al., 2019; Pisarek‐Horowitz et al., 2020). Two glomeruli from each mouse were randomly examined.

### 2.6 Protein assay

The concentrations of hyaluronan in perfusate and serum samples were measured using a hyaluronan enzyme‐linked immunosorbent assay (ELISA) kit (PG Research) according to the instructions from the manufacturer. Proteinuria was measured by BCA assay (Thermo Fisher Scientific). Mice albuminuria and creatinine were determined by mouse urinary albumin assay kit or creatinine ELISA kit (Wako Pure Chemical). The ELISA kits used are listed in Table S1.

### 2.7 Statistical analyses

SPSS version 25.0 software (IBM) was used for statistical analyses. Continuous variables are expressed as mean ± standard deviation or median (interquartile range), as appropriate, and categorical variables are expressed as number (proportion). Differences between variables across samples were assessed by *t*‐test, Wilcoxon rank‐sum test, one‐way analysis of variance (ANOVA), or Kruskal–Wallis test. Post hoc testing after one‐way ANOVA or Kruskal–Wallis testing was performed using Tukey's or Dunn's multiple comparison tests, respectively. Significance was defined at the level of *p *< 0.05.

## 3 RESULTS

### 3.1 Ex vivo rat experiments with the isolated perfusion rat kidney system

**FIGURE 1:**
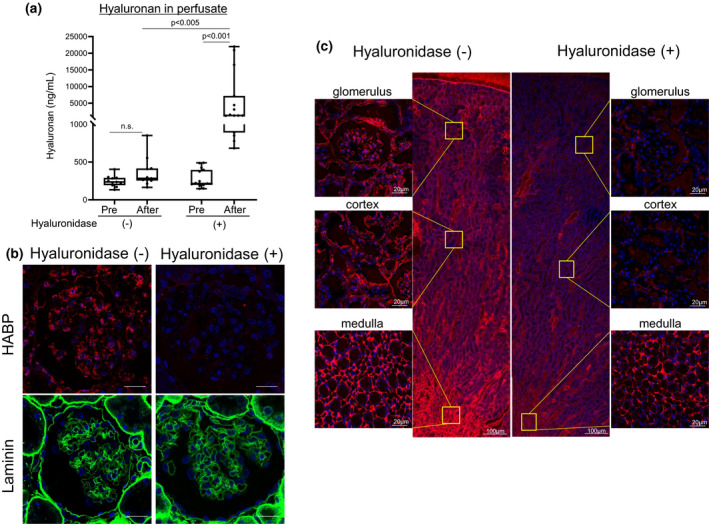
Effects of hyaluronidase in isolated rat kidney. The right renal artery is cannulated and perfused with perfusate for 5 min to wash out blood and to provide an equilibration period. Perfused kidneys are then treated with bolus administration of hyaluronidase (*n* = 15). Control kidneys are administered only perfusate without hyaluronidase (*n* = 11) (Figures S1a, Figure S2a). (a) Hyaluronan levels in perfusate are increased only after administration with hyaluronidase. (b) Hyaluronan expression in glomeruli as evaluated by hyaluronan‐binding protein (HABP) is lost after hyaluronidase administration. Expression of laminin, one of the main components of the glomerular basement membrane, is similarly observed in both groups. (c) Expression of hyaluronan in control and kidney perfused with hyaluronidase. Expression of hyaluronan is prominent in the medulla in control kidney. After hyaluronidase perfusion, hyaluronan expression is lost in the glomerular capillaries and partly disappears in the non‐vascular area. n.s., not significant. Scale bars = 20 μm

We first investigated the effects of hyaluronidase on the degradation of glomerular hyaluronan in isolated perfused kidney (Figure S1a). We measured hyaluronan levels in perfusate before and just after kidney perfusion. Hyaluronan levels were significantly increased after perfusion in the hyaluronidase‐treated group, but no difference was evident before and after kidney perfusion in the control group (Figure 1a). Glomerular hyaluronan expression as assessed by staining for HABP was lost after perfusion with hyaluronidase (Figure 1b). In contrast, expression of laminin, a main component of basement membrane, was similar in both groups (Figure 1b). In the medulla, expression of hyaluronan was prominent in control kidney, but was diminished after hyaluronidase perfusion, although not completely absent (Figure 1b,c). These findings indicate that hyaluronidase treatment successfully degraded hyaluronan on the luminal surface of glomerular endothelial cells.

**FIGURE 2:**
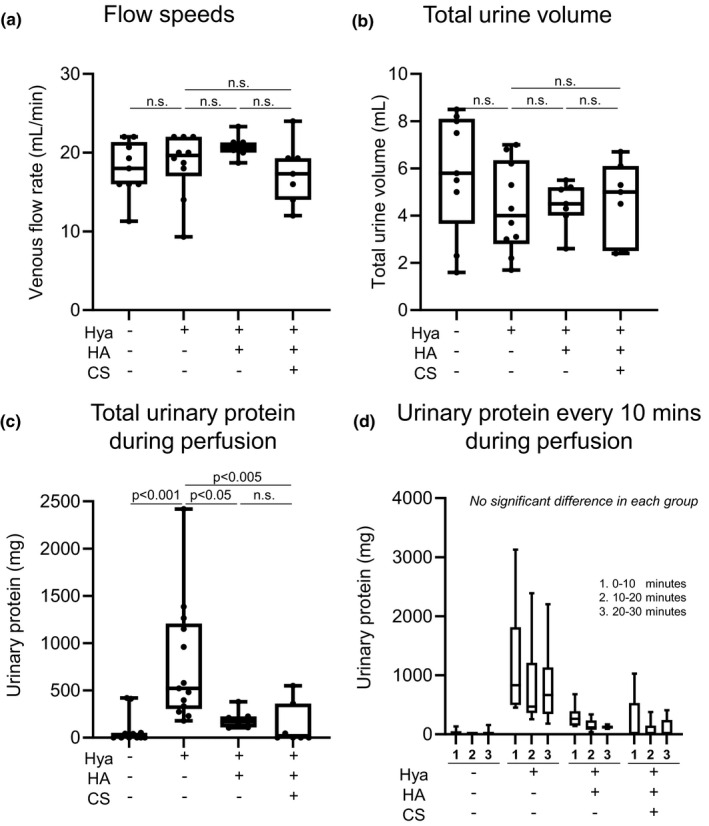
Effects of supplementation with hyaluronan after hyaluronidase digestion in ex vivo. (a) Average flow speeds of perfusate from renal artery to renal vein. (b) Total urine volumes during perfusion. (c) Total urinary protein during perfusion. (d) Urinary protein every 10 min during perfusion. Proteinuria is significantly increased in the hyaluronidase‐treated group (c). Proteinuria is suppressed after supplementation with hyaluronan alone or hyaluronan and chondroitin sulfate (c, d). CS, chondroitin sulfate; HA, hyaluronan; Hya, hyaluronidase; n.s., not significant

Next, we analyzed the effects of urinary protein excretion after degradation of glomerular hyaluronan by hyaluronidase (Figure S1b). No differences in average flow rates of perfusate from renal artery to vein or total urine volumes during perfusion were found between groups (Figure 2a,b). However, the mean amount of urine protein, which mainly consisted of BSA, was significantly increased in the hyaluronidase‐treated group compared with the control group (Figure 2c). Urinary protein excretion rates did not differ during perfusion (10, 20, and 30 min) in each group (Figure 2d). As a next step, we supplemented hyaluronan with or without chondroitin sulfate after hyaluronidase administration to reconstitute glomerular hyaluronan. Notably, proteinuria was dramatically reduced in the kidneys treated with supplemental hyaluronan or hyaluronan and chondroitin sulfate (Figure 2c,d).

### 3.2 Pathological evaluation of perfused kidneys

**FIGURE 3:**
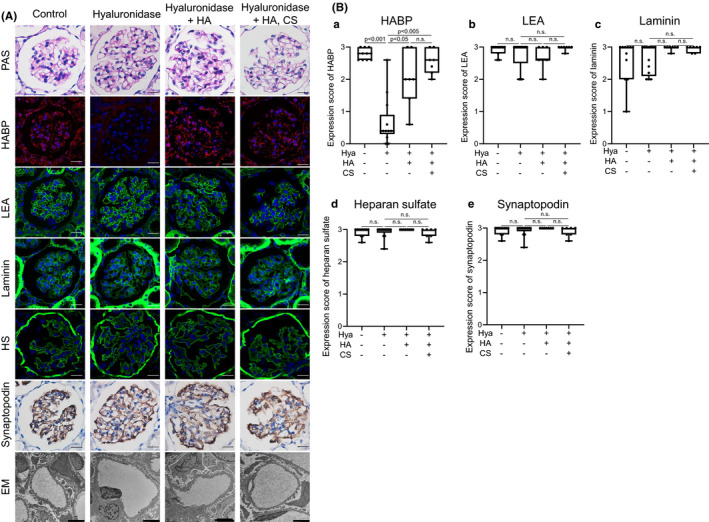
Pathological evaluation of rat kidneys after perfused with IPRK system (ex vivo). (A) Renal tissues after isolated perfused kidney system are shown. Tissues are stained for periodic acid–Schiff (PAS), hyaluronan‐binding protein (HABP), tomato‐derived *Lycopersicon esculentum* lectin (LEA), laminin, heparan sulfate (HS), and synaptopodin. Electronic microscopic (EM) findings are shown. Scale bars except EM = 20 μm, Scale bars in EM = 2.0 μm. (B) Expression score of HABP, LEA, laminin, heparin sulfate, and synaptopodin in rat kidneys after perfusion with the IPRK system (ex vivo). By semi‐quantitative analyses, HABP expression is significantly decreased in the hyaluronidase treatment group (a). No significant differences are observed in glomerular expression scores for LEA, laminin, heparan sulfate, and synaptopodin among groups (b‐e). n.s., not significant

We investigated pathological changes after treatment with hyaluronidase. We did not identify significant changes under light microscopy with PAS staining among the four groups (Figure 3a). Expression of glomerular hyaluronan as assessed by HABP disappeared after hyaluronidase treatment, but was successfully reconstituted in glomeruli after supplementation with hyaluronan or hyaluronan with chondroitin sulfate. Glomerular expressions of LEA, laminin, heparan sulfate, and synaptopodin as a marker of podocytes were similar between groups. In addition, no differences could be detected in podocytes and endothelial cells by EM (Figure 3a). Using semi‐quantitative analyses, HABP expression was significantly suppressed in the hyaluronidase treatment group. In contrast, no significant differences were observed in glomerular expression of LEA, laminin, heparan sulfate, or synaptopodin among the four groups (Figure 3b).

### 3.3 In vivo mouse experiments with hyaluronidase

**FIGURE 4:**
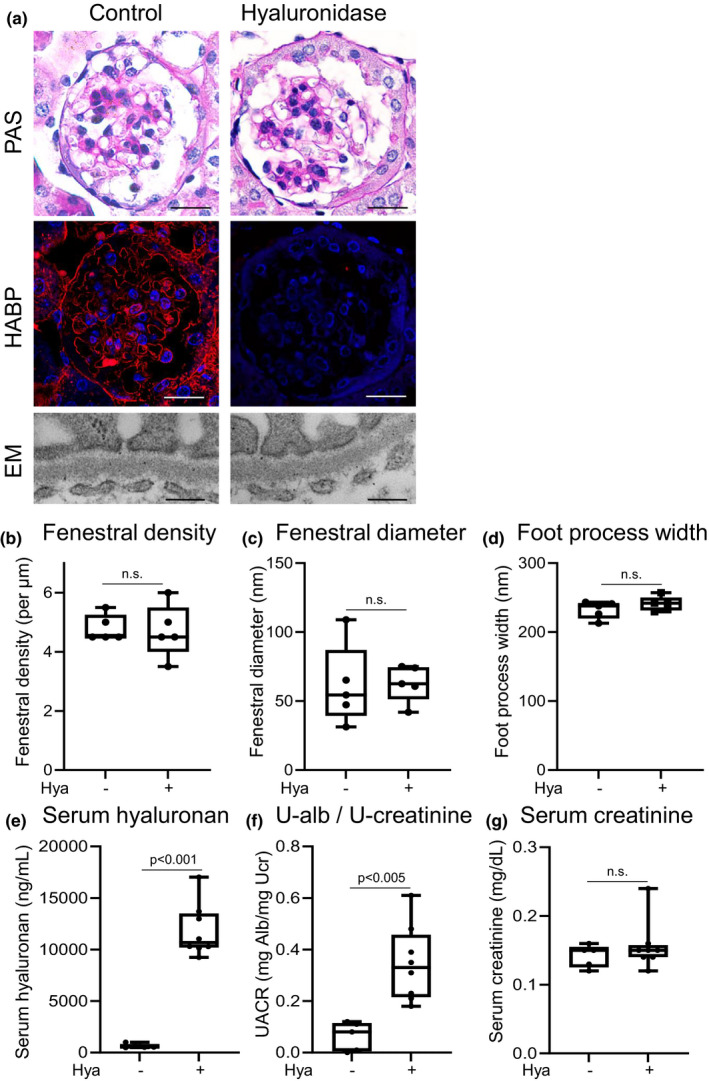
In vivo mouse experiments with hyaluronidase. (a) Pathology (PAS, HABP stain, and EM) of mouse kidney 2 h after injection with or without hyaluronidase. Control mice are injected with saline alone. Scale bars in PAS and HABP stain = 20 μm, Scale bars in EM = 200 nm. (b‐g) Fenestral density, fenestral diameter, foot process width, hyaluronan concentrations in serum, serum creatinine concentration, and albuminuria with and without hyaluronidase administration. Intraperitoneal injection with hyaluronidase (Hya, Hyaluronidase group) increased albuminuria (f) associated with loss of glomerular hyaluronan expression (a) and increase in serum hyaluronan levels (e). No differences are evident in fenestral density (b), fenestral diameter (c), and foot process width (d). n.s., not significant

To investigate the roles of hyaluronan in glomerular endothelial cells in vivo, we further studied the effects of proteinuria by systemic administration of hyaluronidase in mice (Figure S1c). HABP staining demonstrated that hyaluronan expression was decreased in glomeruli and tubules of the hyaluronidase‐treated group (Figure 4a). Two hours after hyaluronidase injection, urinary albumin excretion, which was considered to represent glomerular proteinuria, was significantly increased in association with elevation of serum hyaluronan levels in hyaluronidase‐treated mice compared with non‐treated control mice. No significant differences in serum creatinine levels or morphological structures of endothelial cells, including fenestral density, fenestral diameter, and foot process width, were identified between groups (Figure 4b–g). The results of in vivo experiments were thus congruent with those of ex vivo experiments.

### 3.4 Expression of HABP in human glomerular diseases due to VEGF signaling inhibition

**FIGURE 5:**
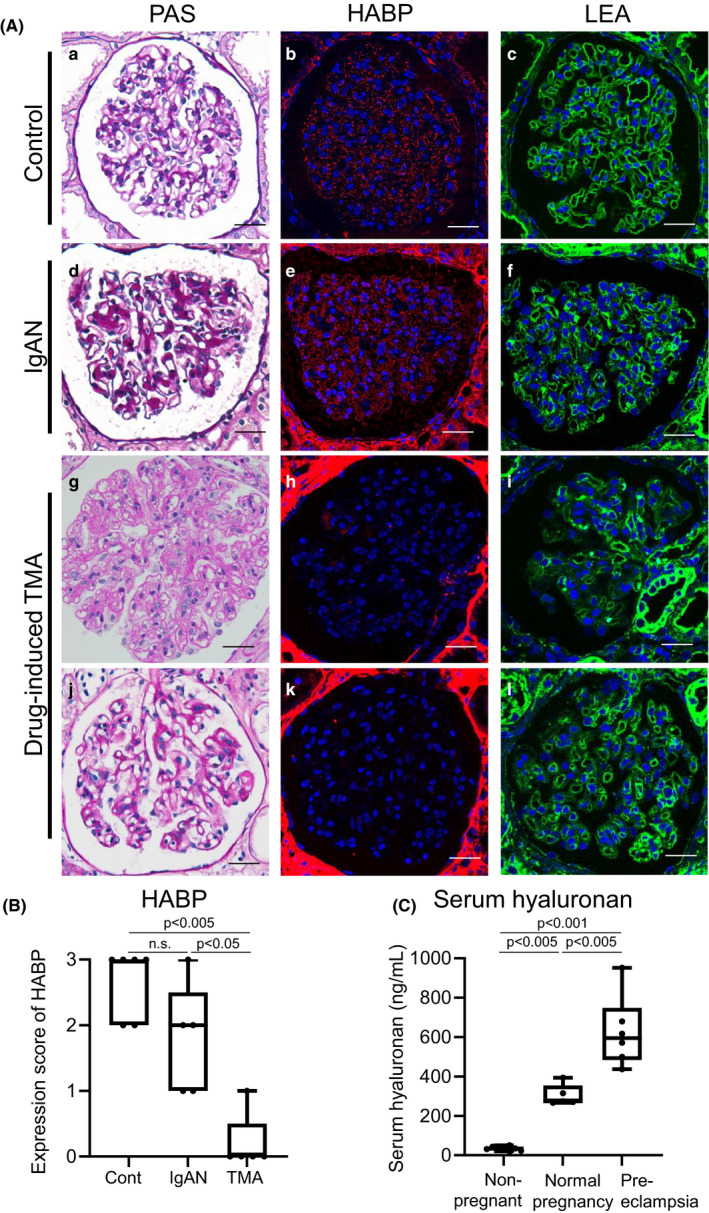
Expression of HABP and serum hyaluronan concentrations in human diseases due to inhibition of VEGF–VEGFR2 signaling. (A) Expression of HABP in human kidney diseases due to inhibition of VEGF–VEGFR2 signaling by anticancer therapy. a, b, c: Control kidney tissues taken at 2 or 3 months after kidney transplantation as protocol biopsies (Cont), d, e, f: IgA nephropathy with mild proteinuria (IgAN), and g to l: Drug‐induced TMA (TMA). a, d, g, j: PAS staining, b, e, h. k: HABP staining, and c, f, i, l: LEA staining. Hyaluronan assessed by HABP expression is detected in glomeruli in control and IgA nephropathy with mild mesangial proliferation. In contrast, in glomeruli with endothelial cell damage of patients with anti‐VEGF–VEGFR2 inhibition by anti‐VEGF monoclonal antibody or tyrosine kinase inhibitors, HABP expression is lost even in glomeruli with mild injury to normal‐appearing structures (j, k). Scale bars = 20 μm. (B) Expression score of HABP. HABP expression score is significantly decreased in the drug‐induced TMA group. (C) Serum concentrations of hyaluronan in patients with pre‐eclampsia. Serum levels of hyaluronan in patients with pre‐eclampsia are significantly higher than those in women of the same age group and pregnant women without pre‐eclampsia. n.s., not significant

We further investigated the expression of hyaluronan in human kidney tissues with endothelial injuries. We chose drug‐induced glomerular endothelial damage, in which VEGF signaling was suppressed. The characteristics of patients are shown in Table 1 and Figure S3b. Three of the five patients showing drug‐induced TMA with proteinuria were treated with tyrosine kinase inhibitors, and the remaining two patients were treated with monoclonal antibodies against VEGF or VEGF receptor. HABP staining was significantly reduced in drug‐induced TMA induced by VEGF inhibition compared to control and IgAN with mild mesangial proliferation (Figure 5a,b; Figure S3). Importantly, in drug‐induced TMA, HABP staining was decreased or even lost in glomeruli with mild abnormalities or normal‐appearing structures on PAS staining (Figure 5aJ‐l). In patients with pre‐eclampsia, known to be associated with VEGF suppression and inflammation, serum hyaluronan concentration was significantly increased when compared with healthy women and normal pregnant group (Figure 5c, Table S2).

**TABLE 1:** Renal biopsy cases evaluated for the expression of HABP Renal biopsy cases include five cases of IgAN, five cases of drug‐induced TMA, and six cases of control kidney specimens taken 2–3 months after renal transplantation

	Control	IgAN	Drug‐induced TMA	*p* value
*n*	6	5	5	
Sex, Men, *n* (%)	4 (67)	3 (60)	4 (80)	0.368
Age, year	53.3 ± 11.4	34.6 ± 10.3	70.8 ± 4.82*	0.001
Daily urine protein, g/day	0.19 ± 0.06	0.59 ± 0.27	6.9 ± 4.48*	0.001
Serum creatinine, mg/dl	1.25 ± 0.17	0.76 ± 0.28*	1.19 ± 0.12	0.004
eGFR, ml/min/1.73 m^2^	45.2 ± 4.96	92.7 ± 25.9*	45.0 ± 4.26	<0.001

Data are presented as number (percentage) or mean ± SD. SD, standard deviation.

* Significant difference compared with control, at a significance level of *p* < 0.05.

## 4 DISCUSSION

**FIGURE 6:**
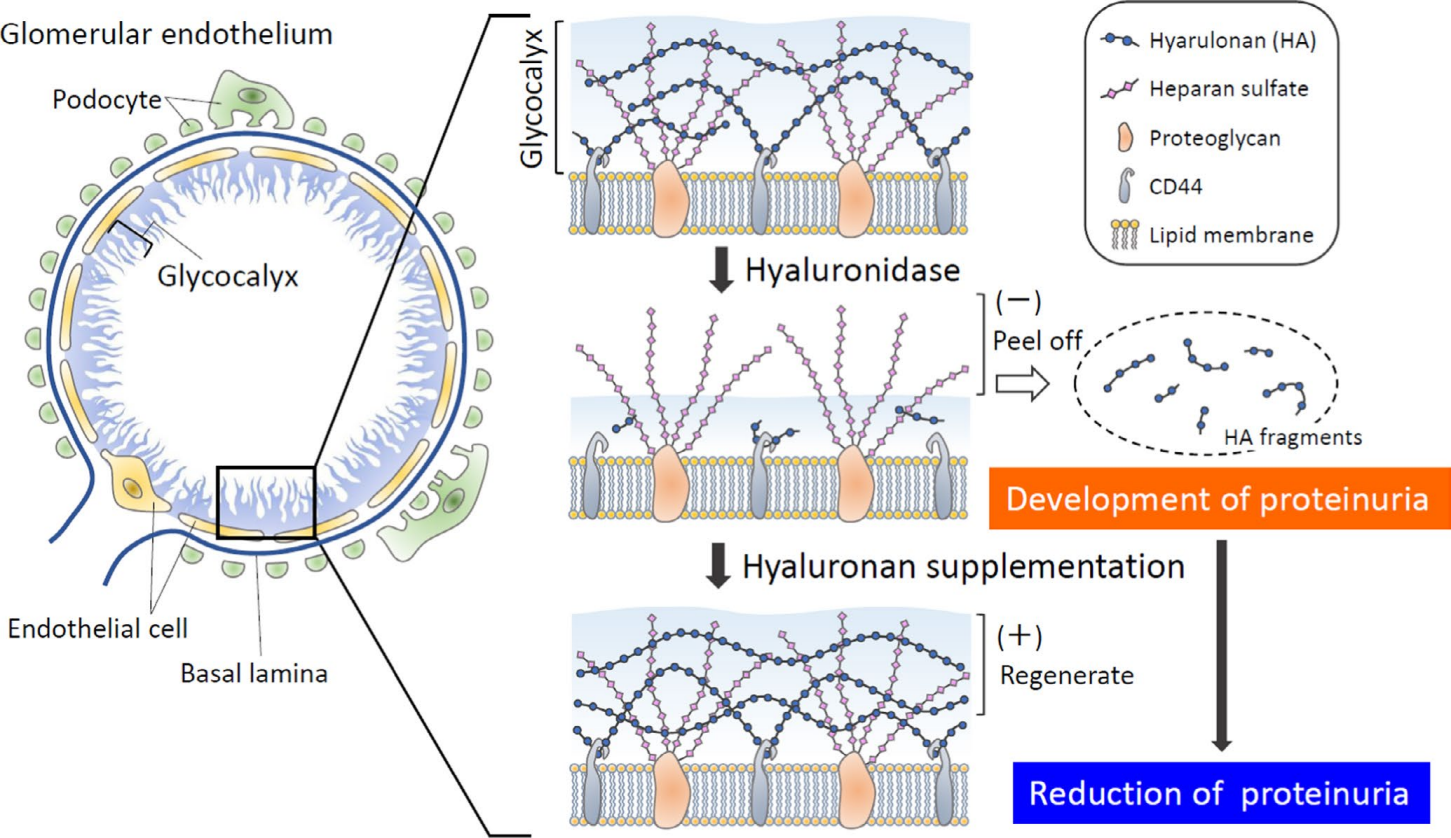
Roles of hyaluronan in glomerular diseases. Surface of glomerular endothelial cells is covered with glycocalyx, a bioactive gel‐like layer composed of heparan sulfate, hyaluronan (HA), chondroitin sulfate, and associated proteins. Hyaluronan is bounded with CD44 in lipid membrane layers. After digestion with hyaluronidase, proteinuria developed in ex vivo and in vivo experiments by destruction of hyaluronan in glomerular endothelial cells. By adding hyaluronan after hyaluronidase, proteinuria is reduced in association with reconstitution of hyaluronan

Glycocalyx is damaged and degraded in a variety of conditions, including sepsis (Aldecoa et al., 2020; Iba & Levy, 2019; Jourde‐Chiche et al., 2019; Song et al., 2018), renal failure (Dane et al., 2014; Jourde‐Chiche et al., 2019; Pletinck et al., 2013), diabetes (Aldecoa et al., 2020; Bogdani et al., 2014; Butler et al., 2020; Dogné & Flamion, 2020; Jourde‐Chiche et al., 2019; Onions et al., 2019; Van Den Berg et al., 2019), and acute respiratory distress syndrome (Butler et al., 2020; Liu et al., 2018; Schmidt et al., 2012). Such degradation may lead to protein leakage and tissue edema (Cosgun et al., 2020; Dogné & Flamion, 2020). This study showed that glomerular hyaluronan plays a crucial role in preventing leakage of proteinuria through glomerular endothelial cells (Figure 6). First, we conducted ex vivo studies to identify the direct effects of hyaluronan in glycocalyx, to exclude the influences of circulating mediators and metabolic disorders, because glycocalyx is degraded by inflammatory diseases and hyperglycemic conditions (Aldecoa et al., 2020; Bogdani et al., 2014; Dane et al., 2014; Diebel et al., 2019; Dogné & Flamion, 2020; Iba & Levy, 2019; Jourde‐Chiche et al., 2019; Liu et al., 2018; Pletinck et al., 2013; Schmidt et al., 2012; Song et al., 2018; Van Den Berg et al., 2019) (Figures 1 and 2). We established ex vivo experiments (Figure 2) that showed similar experimental conditions in venous flow rates and urine volumes among groups (Chang et al., 2013). We detected increased hyaluronan in the perfusate (Figure 1A) or serum (Figure 4E) after hyaluronidase treatment, suggesting that glomerular hyaluronan was degraded and serum levels of hyaluronan can provide a biomarker for the destruction of endothelial glycocalyx as reported under septic conditions (Yagmur et al., 2012). In previous studies, the presence or loss of glycocalyx was judged by the thickness measured using sophisticated techniques (Desideri et al., 2018; Henry & Duling, 1999; Jeansson & Haraldsson, 2003; Potter & Damiano, 2008; Vink & Duling, 1996). The present study successfully demonstrated the presence of hyaluronan in the glomerular endothelial surface layers by detection of biotin‐labeled HABP using Maskedform fixed paraffin‐embedded sections. In a recent report, fixation was found to alter the ability to detect hyaluronan in tissue microstructures (Rowley et al., 2020). The procedures in our experiments enabled visualization of the presence of hyaluronan in glomeruli. Proteinuria developed in the ex vivo system after digestion of hyaluronan in the glomerular capillaries (Figures 2 and 3). Furthermore, supplementation with hyaluronan successfully suppressed proteinuria (Figure 2), suggesting hyaluronan as a plausible therapeutic target. We provided supplementation with a mixture of hyaluronan and chondroitin sulfate after hyaluronidase treatment, because previous studies have suggested that hyaluronan alone cannot reconstitute hyaluronan in an assessment of thickness of the glycocalyx layer (Henry & Duling, 1999; Potter & Damiano, 2008). Addition of exogenous hyaluronan alone reportedly failed to be incorporated into the glycocalyx unless another glycosaminoglycan is present (Henry & Duling, 1999; Potter & Damiano, 2008). In the present in vivo study, mice successfully developed albuminuria after intraperitoneal injection with hyaluronidase associated with loss of glomerular hyaluronan (Figure 4). These findings indicate that hyaluronan contributes to preventing proteinuria from leakage and preserving the integrity of endothelial cells.

Several antitumor therapeutics targeting the angiogenic VEGF signaling pathway have been widely applied for a variety of cancers (Estrada et al., 2019). Anti‐VEGF–VEGFR2 drugs are well known to cause TMA in association with proteinuria, as so‐called anti‐VEGF therapy‐induced “glomerular microangiopathy” (Pfister et al., 2018), and in some cases dialysis therapies are required (Vigneau et al., 2014). In our pathological analyses, HABP staining was decreased in these conditions even in normal appearing to mildly injured glomeruli, suggesting that shedding of hyaluronan may occur from a relatively early state in these pathological conditions. Fortunately, renal function in these patients was preserved after stopping anti‐VEGF therapy (Figure S3C). In pre‐eclampsia with endothelial dysfunctions, another condition under VEGF signaling inhibition (Butler et al., 2020; Jourde‐Chiche et al., 2019; Levine et al., 2004; Moghaddas Sani et al., 2019) due to anti‐angiogenic factor, soluble fms‐like tyrosine kinase 1 produced by diseased placenta, and inflammation, serum levels of hyaluronan were significantly increased in our analyses (Figure 5C), supporting previous reports (Weissgerber et al., 2019; Ziganshina et al., 2020). Our findings suggest the importance of hyaluronan in the development of human kidney diseases with endothelial dysfunctions due to inhibition of VEGF signaling.

Recently, glycocalyx has been reported to be involved in cross talk between podocytes and glomerular endothelial cells (Ebefors et al., 2019; Fu et al., 2015; Jourde‐Chiche et al., 2019). Hyaluronan in glycocalyx is strongly suggested to play roles in the development of diabetic complications (Dogné & Flamion, 2020; Rabelink & De Zeeuw, 2015; Van Den Berg et al., 2019). Knockout mice lacking endothelial hyaluronan synthase 2 showed a loss of glomerular endothelial hyaluronan leading to mesangiolysis, secondary podocytes injuries, and glomerulosclerosis (Van Den Berg et al., 2019). Biopsy specimen from patients with diabetic nephropathy showed a loss of glomerular endothelial hyaluronan in association with structural damage (Van Den Berg et al., 2019). On the other hand, podocyte activation and pathologic cross talk with endothelial cells via endothelin‐1 were recently reported to induce dysfunction and loss of endothelial glycocalyx leading to albuminuria (Ebefors et al., 2019). Our data support those findings and suggest that loss of glomerular hyaluronan seems important in the development of glomerular diseases with endothelial injury by direct or podocyte–endothelial cross talk.

In summary, vascular endothelial cells have several factors and functions for protecting against injury and dysfunction. Our data suggest that hyaluronan itself, as one of the components of glycocalyx, plays a crucial role in preventing proteinuria and preserving the integrity of endothelial cells. Hyaluronan could be a therapeutic target for ameliorating glomerular endothelial glycocalyx damage, including under conditions of VEGF signaling inhibition and inflammation.

## CONFLICT OF INTEREST

None declared.

## AUTHOR CONTRIBUTION

All the authors contributed to this study and approved the final version of the manuscript. A.A., N.H., S.H., M.N., and Y.I. conceived and designed the study. A.A, K.K., M. Yamauchi, H.K., and N.H. carried out the experiments. K.W., A.W., S.M., and T.I. collected the clinical samples. M. Yamaguchi and T.K. performed the statistical analysis. H.N., J.A., T.I, S.H., M.N., and Y.I. contributed to the interpretation of data. A.A., H.N., and Y.I. drafted the manuscript. M. Yamaguchi, N.H., A.W., J.A., S.M., T.I, S.H., and M.N. provided critical feedback and commented the manuscript.

## Supporting information



Supplementary Material

## References

[phy215019-bib-0001] Aldecoa, C., Llau, J. V., Nuvials, X., & Artigas, A. (2020). Role of albumin in the preservation of endothelial glycocalyx integrity and the microcirculation: A review. Ann Intensive Care, 10, 1–12. 32572647 10.1186/s13613-020-00697-1 PMC7310051

[phy215019-bib-0002] Bogdani, M., Johnson, P. Y., Potter‐Perigo, S., Nagy, N., Day, A. J., Bollyky, P. L., & Wight, T. N. (2014). Hyaluronan and hyaluronan‐binding proteins accumulate in both human type 1 diabetic islets and lymphoid tissues and associate with inflammatory cells in insulitis. Diabetes, 63, 2727–2743. 24677718 10.2337/db13-1658 PMC4113060

[phy215019-bib-0003] Brinkkoetter, P. T., Ising, C., & Benzing, T. (2013). The role of the podocyte in albumin filtration. Nature Reviews Nephrology, 9, 328–336. 23609563 10.1038/nrneph.2013.78

[phy215019-bib-0004] Brocklebank, V., Wood, K. M., & Kavanagh, D. (2018). Thrombotic microangiopathy and the kidney. Clinical Journal of the American Society of Nephrology, 13, 300–317. 29042465 10.2215/CJN.00620117 PMC5967417

[phy215019-bib-0005] Butler, M. J., Down, C. J., Foster, R. R., & Satchell, S. C. (2020). The pathological relevance of increased endothelial glycocalyx permeability. American Journal of Pathology, 190, 742–751. 10.1016/j.ajpath.2019.11.015 PMC7163249 32035881

[phy215019-bib-0006] Chang, H., Choong, B., Phillips, A., & Loomes, K. M. (2013). The isolated perfused rat kidney: A technical update. Experimental Animals, 62, 19–23. 23357942 10.1538/expanim.62.19

[phy215019-bib-0007] Cosgun, Z. C., Fels, B., & Kusche‐Vihrog, K. (2020). Nanomechanics of the endothelial glycocalyx: From structure to function. American Journal of Pathology, 190, 732–741. 10.1016/j.ajpath.2019.07.021 32035884

[phy215019-bib-0008] Dane, M. J. C., Khairoun, M. R., Hyun Lee, D., van den Berg, B. M., Eskens, B. J. M., Boels, M. G. S., van Teeffelen, J. W. G. E., Rops, A. L. W. M. M., van der Vlag, J. V., van Zonneveld, A. J., Reinders, M. E. J., Vink, H., & Rabelink, T. J. (2014). Association of kidney function with changes in the endothelial surface layer. Clinical Journal of the American Society of Nephrology, 9, 698–704. 24458084 10.2215/CJN.08160813 PMC3974363

[phy215019-bib-0009] Dane, M. J. C., van den Berg, B. M., Avramut, M. C., Faas, F. G. A., van der Vlag, J., Rops, A. L. W. M. M., Ravelli, R. B. G., Koster, B. J., van Zonneveld, A. J., Vink, H., & Rabelink, T. J. (2013). Glomerular endothelial surface layer acts as a barrier against albumin filtration. American Journal of Pathology, 182, 1532–1540. 10.1016/j.ajpath.2013.01.049 23518410

[phy215019-bib-0010] Dane, M. J. C., Van Den Berg, B. M., Lee, D. H., Boels, M. G. S., Tiemeier, G. L., Avramut, M. C., Van Zonneveld, A. J., Van Der Vlag, J., Vink, H., & Rabelink, T. J. (2015). A microscopic view on the renal endothelial glycocalyx. American Journal of Physiology. Renal Physiology, 308, 956–966. 10.1152/ajprenal.00532.2014 25673809

[phy215019-bib-0011] Desideri, S., Onions, K. L., Qiu, Y., Ramnath, R. D., Butler, M. J., Neal, C. R., King, M. L. R., Salmon, A. E., Saleem, M. A., Welsh, G. I., Michel, C. C., Satchell, S. C., Salmon, A. H. J., & Foster, R. R. (2018). A novel assay provides sensitive measurement of physiologically relevant changes in albumin permeability in isolated human and rodent glomeruli. Kidney International, 93, 1086–1097. 29433915 10.1016/j.kint.2017.12.003 PMC5912930

[phy215019-bib-0012] Diebel, L. N., Liberati, D. M., & Martin, J. V. (2019). Acute hyperglycemia increases sepsis related glycocalyx degradation and endothelial cellular injury: A microfluidic study. American Journal of Surgery, 217, 1076–1082. 30635208 10.1016/j.amjsurg.2018.12.066

[phy215019-bib-0013] Dogné, S., & Flamion, B. (2020). Endothelial glycocalyx impairment in disease: Focus on hyaluronan shedding. American Journal of Pathology, 190, 768–780. 10.1016/j.ajpath.2019.11.016 32035885

[phy215019-bib-0014] Ebefors, K., Wiener, R. J., Yu, L., Azeloglu, E. U., Yi, Z., Jia, F., Zhang, W., Baron, M. H., He, J. C., Haraldsson, B., & Daehn, I. (2019). Endothelin receptor‐A mediates degradation of the glomerular endothelial surface layer via pathologic crosstalk between activated podocytes and glomerular endothelial cells. Kidney International, 96, 957–970. 31402170 10.1016/j.kint.2019.05.007 PMC7200072

[phy215019-bib-0015] Estrada, C. C., Maldonado, A., & Mallipattu, S. K. (2019). Therapeutic inhibition of VEGF signaling and associated nephrotoxicities. Journal of the American Society of Nephrology, 30, 187–200. 30642877 10.1681/ASN.2018080853 PMC6362621

[phy215019-bib-0016] Fissell, W. H., & Miner, J. H. (2018). What is the glomerular ultrafiltration barrier? Journal of the American Society of Nephrology, 29, 2262–2264. 30030419 10.1681/ASN.2018050490 PMC6115656

[phy215019-bib-0017] Fu, J., Lee, K., Chuang, P. Y., Liu, Z., & He, J. C. (2015). Glomerular endothelial cell injury and cross talk in diabetic kidney disease. American Journal of Physiology. Renal Physiology, 308, 287–297. 10.1152/ajprenal.00533.2014 PMC4329492 25411387

[phy215019-bib-0018] Henry, C. B. S., & Duling, B. R. (1999). Permeation of the luminal capillary glycocalyx is determined by hyaluronan. American Journal of Physiology. Heart and Circulatory Physiology, 277, 508–514. 10.1152/ajpheart.1999.277.2.H508 10444475

[phy215019-bib-0019] Iba, T., & Levy, J. H. (2019). Derangement of the endothelial glycocalyx in sepsis. Journal of Thrombosis and Haemostasis, 17, 283–294. 30582882 10.1111/jth.14371

[phy215019-bib-0020] Jeansson, M., & Haraldsson, B. (2003). Glomerular size and charge selectivity in the mouse after exposure to glucosaminoglycan‐degrading enzymes. Journal of the American Society of Nephrology, 14, 1756–1765. 12819235 10.1097/01.asn.0000072742.02714.6e

[phy215019-bib-0021] Jeansson, M., & Haraldsson, B. (2006). Morphological and functional evidence for an important role of the endothelial cell glycocalyx in the glomerular barrier. American Journal of Physiology, Renal Physiology, 290, 111–116. 10.1152/ajprenal.00173.2005 16091582

[phy215019-bib-0022] Jourde‐Chiche, N., Fakhouri, F., Dou, L., Bellien, J., Burtey, S., Frimat, M., Jarrot, P. A., Kaplanski, G., Le Quintrec, M., Pernin, V., Rigothier, C., Sallée, M., Fremeaux‐Bacchi, V., Guerrot, D., & Roumenina, L. T. (2019). Endothelium structure and function in kidney health and disease. Nature Reviews Nephrology, 15, 87–108. 30607032 10.1038/s41581-018-0098-z

[phy215019-bib-0023] Katsuno, T., Ito, Y., Kagami, S., Kitamura, H., Maruyama, S., Shimizu, A., Sugiyama, H., Sato, H., Yokoyama, H., & Kashihara, N. (2020). A nationwide cross‐sectional analysis of thrombotic microangiopathy in the Japan Renal Biopsy Registry (J‐RBR). Clinical and Experimental Nephrology, 24, 789–797. 32415379 10.1007/s10157-020-01896-7

[phy215019-bib-0024] Kinashi, H., Ito, Y., Mizuno, M., Suzuki, Y., Terabayashi, T., Nagura, F., Hattori, R., Matsukawa, Y., Mizuno, T., Noda, Y., Nishimura, H., Nishio, R., Maruyama, S., Imai, E., Matsuo, S., & Takei, Y. (2013). TGF‐β1 promotes lymphangiogenesis during peritoneal fibrosis. Journal of the American Society of Nephrology, 24, 1627–1642. 23990681 10.1681/ASN.2012030226 PMC3785267

[phy215019-bib-0025] Levine, R. J., Maynard, S. E., Qian, C., Lim, K.‐H., England, L. J., Yu, K. F., Schisterman, E. F., Thadhani, R., Sachs, B. P., Epstein, F. H., Sibai, B. M., Sukhatme, V. P., & Karumanchi, S. A. (2004). Circulating angiogenic factors and the risk of preeclampsia. New England Journal of Medicine, 350, 672–683. 10.1056/NEJMoa031884 14764923

[phy215019-bib-0026] Liu, X. Y., Xu, H. X., Li, J. K., Zhang, D., Ma, X. H., Huang, L. N., Lü, J. H., & Wang, X. Z. (2018). Neferine protects endothelial glycocalyx via mitochondrial ROS in lipopolysaccharide‐induced acute respiratory distress syndrome. Frontiers in Physiology, 9, 1–14. 29520236 10.3389/fphys.2018.00102 PMC5826949

[phy215019-bib-0027] Moghaddas Sani, H., Zununi Vahed, S., & Ardalan, M. (2019). Preeclampsia: A close look at renal dysfunction. Biomedicine & Pharmacotherapy, 109, 408–416. 30399576 10.1016/j.biopha.2018.10.082

[phy215019-bib-0028] Oltean, S., Qiu, Y., Ferguson, J. K., Stevens, M., Neal, C., Russell, A., Kaura, A., Arkill, K. P., Harris, K., Symonds, C., Lacey, K., Wijeyaratne, L., Gammons, M., Wylie, E., Hulse, R. P., Alsop, C., Cope, G., Damodaran, G., Betteridge, K. B., … Salmon, A. H. J. (2015). Vascular endothelial growth factor‐A165b is protective and restores endothelial glycocalyx in diabetic nephropathy. Journal of the American Society of Nephrology, 26, 1889–1904. 25542969 10.1681/ASN.2014040350 PMC4520162

[phy215019-bib-0029] Onions, K. L., Gamez, M., Buckner, N. R., Baker, S. L., Betteridge, K. B., Desideri, S., Dallyn, B. P., Ramnath, R. D., Neal, C. R., Farmer, L. K., Mathieson, P. W., Gnudi, L., Alitalo, K., Bates, D. O., Salmon, A. H. J., Welsh, G. I., Satchell, S. C., & Foster, R. R. (2019). VEGFC reduces glomerular albumin permeability and protects against alterations in VEGF receptor expression in diabetic nephropathy. Diabetes, 68, 172–187. 30389746 10.2337/db18-0045

[phy215019-bib-0030] Pfister, F., Amann, K., Daniel, C., Klewer, M., Büttner, A., & Büttner‐Herold, M. (2018). Characteristic morphological changes in anti‐VEGF therapy‐induced glomerular microangiopathy. Histopathology, 73, 990–1001. 30014486 10.1111/his.13716

[phy215019-bib-0031] Pisarek‐Horowitz, A., Fan, X., Kumar, S., Rasouly, H. M., Sharma, R., Chen, H., Coser, K., Bluette, C. T., Hirenallur‐Shanthappa, D., Anderson, S. R., Yang, H., Beck, L. H., Bonegio, R. G., Henderson, J. M., Berasi, S. P., Salant, D. J., & Lu, W. (2020). Loss of Roundabout Guidance Receptor 2 (Robo2) in podocytes protects adult mice from glomerular injury by maintaining podocyte foot. American Journal of Pathology, 190, 799–816. 10.1016/j.ajpath.2019.12.009 PMC7217334 32220420

[phy215019-bib-0032] Pletinck, A., Glorieux, G., Schepers, E., Cohen, G., Gondouin, B., Van Landschoot, M., Eloot, S., Rops, A., Van De Voorde, J., De Vriese, A., Van Der Vlag, J., Brunet, P., Van Biesen, W., & Vanholder, R. (2013). Protein‐bound uremic toxins stimulate crosstalk between leukocytes and vessel wall. Journal of the American Society of Nephrology, 24, 1981–1994. 24009240 10.1681/ASN.2012030281 PMC3839540

[phy215019-bib-0033] Potter, D. R., & Damiano, E. R. (2008). The hydrodynamically relevant endothelial cell glycocalyx observed in vivo is absent in vitro. Circulation Research, 102, 770–776. 18258858 10.1161/CIRCRESAHA.107.160226

[phy215019-bib-0034] Rabelink, T. J., & De Zeeuw, D. (2015). The glycocalyx — Linking albuminuria with renal and cardiovascular disease. Nature Reviews Nephrology, 11, 667–676. 26460356 10.1038/nrneph.2015.162

[phy215019-bib-0035] Robertson, R. T., Levine, S. T., Haynes, S. M., Gutierrez, P., Baratta, J. L., Tan, Z., & Longmuir, K. J. (2014). Use of labeled tomato lectin for imaging vasculature structures. Histochemistry and Cell Biology, 143, 225–234. 25534591 10.1007/s00418-014-1301-3

[phy215019-bib-0036] Rowley, J. E., Rubenstein, G. E., Manuel, S. L., Johnson, N. L., Surgnier, J., Kapitsinou, P. P., Duncan, F. E., & Pritchard, M. T. (2020). Tissue‐specific fixation methods are required for optimal in situ visualization of hyaluronan in the ovary, kidney, and liver. Journal of Histochemistry and Cytochemistry, 68, 75–91. 31714169 10.1369/0022155419884879 PMC6931168

[phy215019-bib-0037] Schmidt, E. P., Yang, Y., Janssen, W. J., Gandjeva, A., Perez, M. J., Barthel, L., Zemans, R. L., Bowman, J. C., Koyanagi, D. E., Yunt, Z. X., Smith, L. P., Cheng, S. S., Overdier, K. H., Thompson, K. R., Geraci, M. W., Douglas, I. S., Pearse, D. B., & Tuder, R. M. (2012). The pulmonary endothelial glycocalyx regulates neutrophil adhesion and lung injury during experimental sepsis. Nature Medicine, 18, 1217–1223. 10.1038/nm.2843 PMC3723751 22820644

[phy215019-bib-0038] Singh, A., Satchell, S. C., Neal, C. R., McKenzie, E. A., Tooke, J. E., & Mathieson, P. W. (2007). Glomerular endothelial glycocalyx constitutes a barrier to protein permeability. Journal of the American Society of Nephrology, 18, 2885–2893. 17942961 10.1681/ASN.2007010119

[phy215019-bib-0039] Song, J. W., Zullo, J., Lipphardt, M., Dragovich, M., Zhang, F. X., Fu, B., & Goligorsky, M. S. (2018). Endothelial glycocalyx‐the battleground for complications of sepsis and kidney injury. Nephrology, Dialysis, Transplantation, 33, 203–211. 10.1093/ndt/gfx076 PMC5837430 28535253

[phy215019-bib-0040] Sun, T., Sakata, F., Ishii, T., Tawada, M., Suzuki, Y., Kinashi, H., Katsuno, T., Takei, Y., Maruyama, S., Mizuno, M., & Ito, Y. (2019). Excessive salt intake increases peritoneal solute transport rate via local tonicity‐responsive enhancer binding protein in subtotal nephrectomized mice. Nephrology, Dialysis, Transplantation, 34, 2031–2042. 10.1093/ndt/gfz045 30897196

[phy215019-bib-0041] Suzuki, Y., Ito, Y., Mizuno, M., Kinashi, H., Sawai, A., Noda, Y., Mizuno, T., Shimizu, H., Fujita, Y., Matsui, K., Maruyama, S., Imai, E., Matsuo, S., & Takei, Y. (2012). Transforming growth factor‐b induces vascular endothelial growth factor‐C expression leading to lymphangiogenesis in rat unilateral ureteral obstruction. Kidney International, 81, 865–879. 22258325 10.1038/ki.2011.464

[phy215019-bib-0042] Van Den Berg, B. M., Wang, G., Boels, M. G. S., Avramut, M. C., Jansen, E., Sol, W. M. P. J., Lebrin, F., Van Zonneveld, A. J., De Koning, E. J. P., Vink, H., Gröne, H. J., Carmeliet, P., Van Der Vlag, J., & Rabelink, T. J. (2019). Glomerular function and structural integrity depend on hyaluronan synthesis by glomerular endothelium. Journal of the American Society of Nephrology, 30, 1886–1897. 31308073 10.1681/ASN.2019020192 PMC6779367

[phy215019-bib-0043] Vigneau, C., Lorcy, N., Dolley‐Hitze, T., Jouan, F., Arlot‐Bonnemains, Y., Laguerre, B., Verhoest, G., Goujon, J. M., Belaud‐Rotureau, M. A., & Rioux‐Leclercq, N. (2014). All anti‐vascular endothelial growth factor drugs can induce “pre‐eclampsia‐like syndrome”: A RARe study. Nephrology, Dialysis, Transplantation, 29, 325–332. 10.1093/ndt/gft465 24302609

[phy215019-bib-0044] Vink, H., & Duling, B. R. (1996). Identification of distinct luminal domains for macromolecules, erythrocytes, and leukocytes within mammalian capillaries. Circulation Research, 79, 581–589. 8781491 10.1161/01.res.79.3.581

[phy215019-bib-0045] Weissgerber, T. L., Garcia‐Valencia, O., Milic, N. M., Codsi, E., Cubro, H., Nath, M. C., White, W. M., Nath, K. A., & Garovic, V. D. (2019). Early onset preeclampsia is associated with glycocalyx degradation and reduced microvascular perfusion. Journal of the American Heart Association, 8, 1–11. 10.1161/JAHA.118.010647 PMC6405679 30764695

[phy215019-bib-0046] Yagmur, E., Koch, A., Haumann, M., Kramann, R., Trautwein, C., & Tacke, F. (2012). Hyaluronan serum concentrations are elevated in critically ill patients and associated with disease severity. Clinical Biochemistry, 45, 82–87. 22085533 10.1016/j.clinbiochem.2011.10.016

[phy215019-bib-0047] Ziganshina, M. M., Yarotskaya, E. L., Bovin, N. V., Pavlovich, S. V., & Sukhikh, G. T. (2020). Can endothelial glycocalyx be a major morphological substrate in pre‐eclampsia? International Journal of Molecular Sciences, 21, 3048. 10.3390/ijms21093048 PMC7246531 32357469

