# Supplementary material for: Roles of glomerular endothelial hyaluronan in the development of proteinuria
Source: Physiol Rep. 2021 Sep 2;9(17):e15019. doi: 10.14814/phy2.15019 (PMC8411502; doi:10.14814/phy2.15019)
Supplement: Supplementary file 1 — Supplementary Material [file PHY2-9-e15019-s001.pdf]

Supplementary materials

Roles of glomerular endothelial hyaluronan in the development of proteinuria

Authors:
Akimasa Asai, Naoyuki Hatayama, Keisuke Kamiya, Mai Yamauchi, Hiroshi Kinashi, Makoto Yamaguchi, Takayuki Katsuno, Hironobu Nobata, Kazushi Watanabe, Akihiko Wakatsuki, Jan Aten, Shoichi Maruyama, Takuji Ishimoto, Shuichi Hirai, Munekazu Naito, Yasuhiko Ito

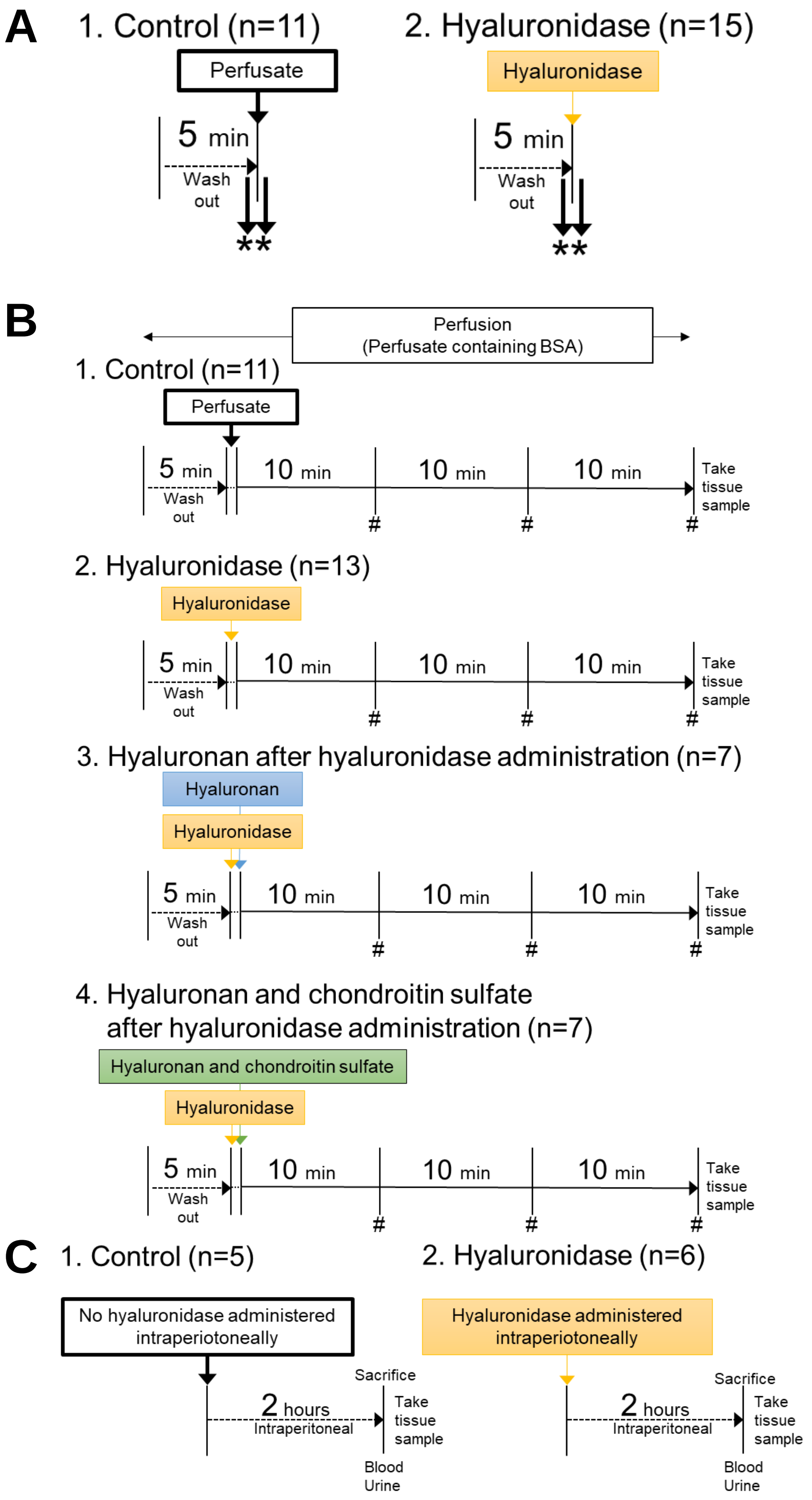

Supplementary Figure 1: Animal experiment protocols
* indicates sample collection of perfusate.
# indicates measurement of urine volume and sample collection of urine.

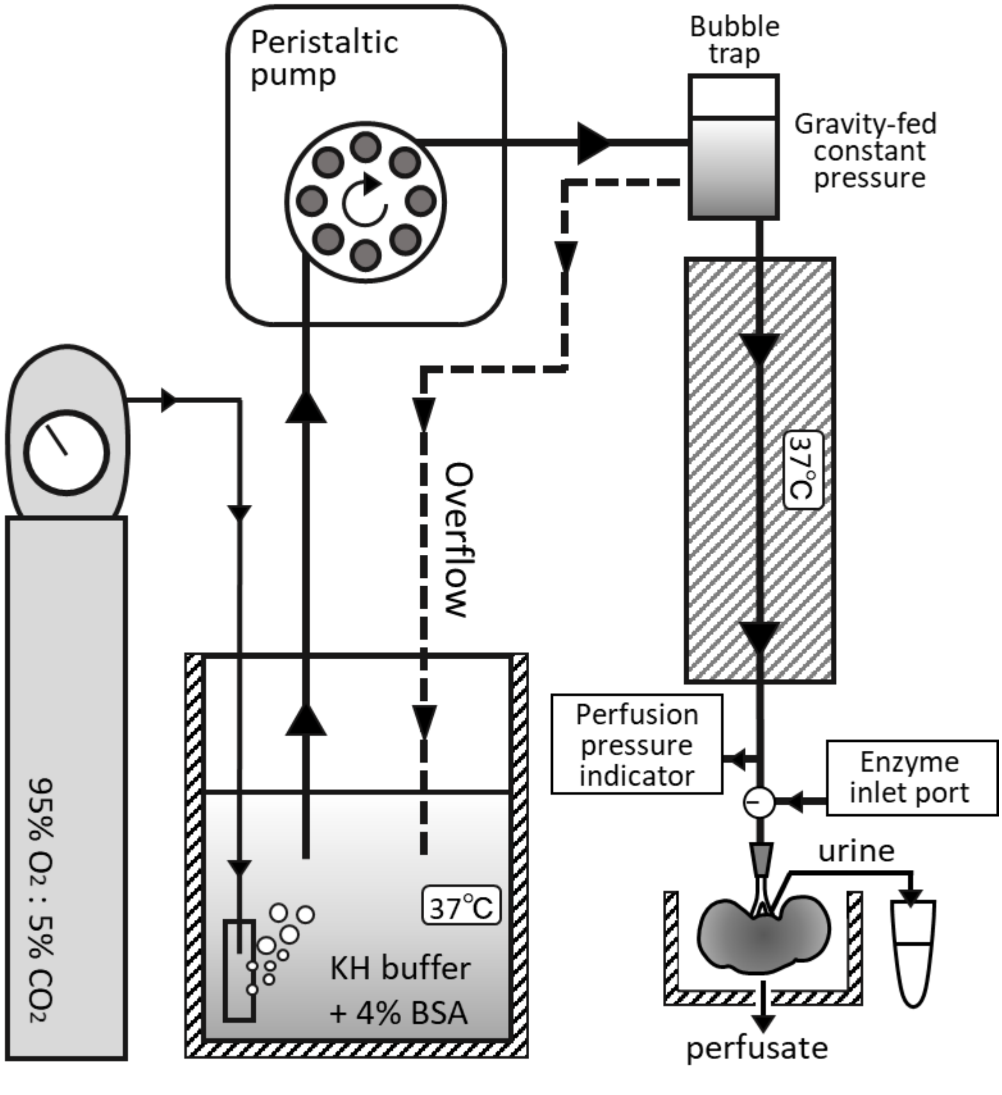

Supplementary Figure 2: Isolated perfusion rat kidney (IPRK) system
KH: Krebs-Henseleit

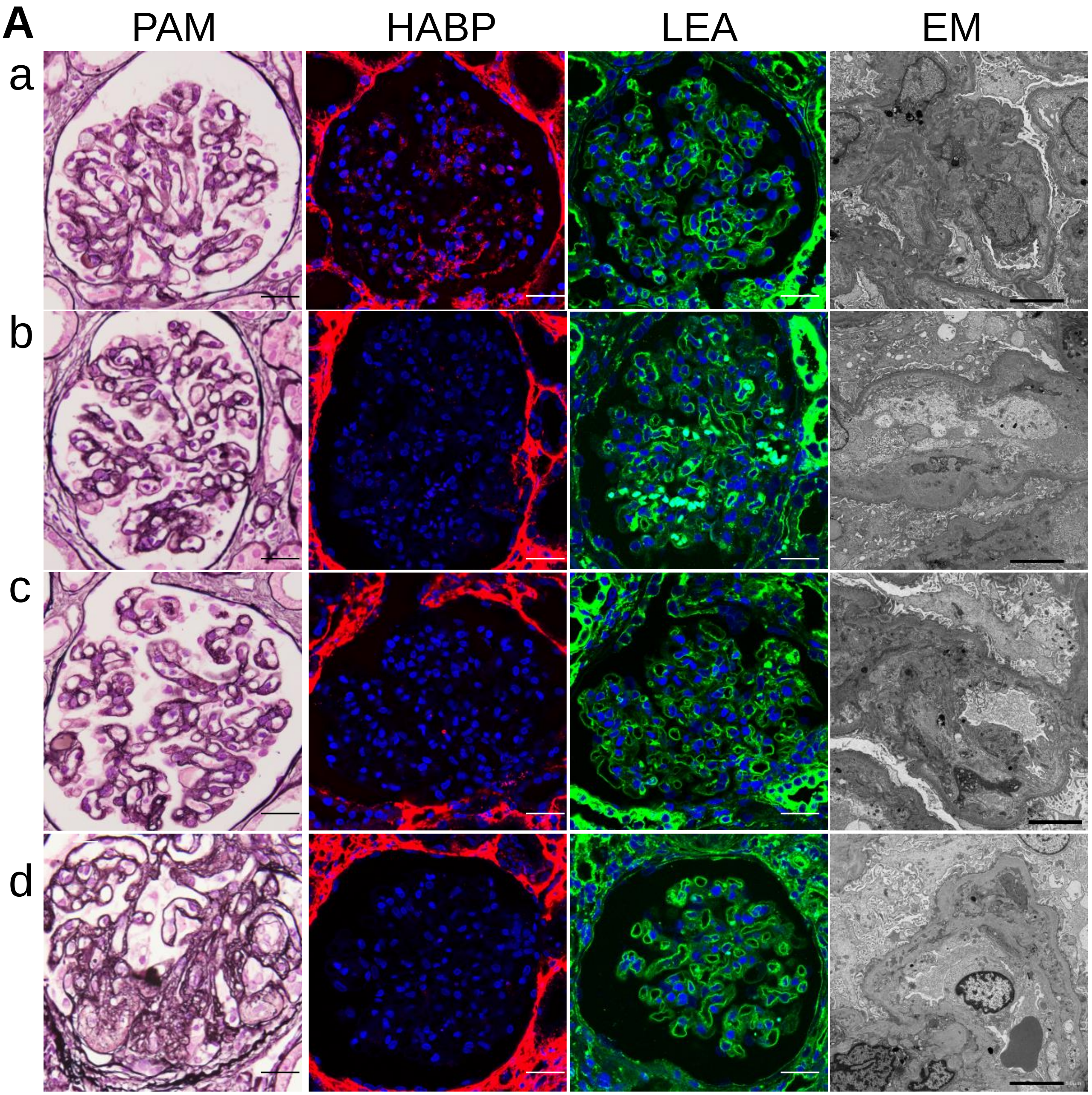

Supplementary Figure 3-A: Expression of HABP in all cases of anti-VEGF therapy-induced glomerular microangiopathy other than a case shown in Figure 6

B Characteristic of biopsy patients treated with anti-VEGF therapy

	Cause	Age	Sex	UP/CR (g/gCr)	Serum creatinine (mg/dL)	eGFR (mL/min/1.73m^2)
Fig5, A g-l	Multitargeted TKI (sunitinib)	72	man	6.89	1.31	42.3
a	Multitargeted TKI (nintedanib)	73	man	6.89	1.13	49.5
b	Multitargeted TKI (lenvatinib)	65	man	1.95	1.16	49.8
c	mAb against VEGFR2 (ramucirumab)	77	man	3.71	1.3	41.9
d	mAb against VEGFA (bevacizumab)	67	woman	8.83	1.03	41.5

C Renal prognosis of patients treated with anti-VEGF therapy

	Change of UP/CR (g/gCr)			Change of eGFR (mL/min/1.73m2)			Life expectancy
	3 months	6 months	Final data (months after biopsy)	3 months	6 months	Final data (months after biopsy)	Alive or death (months after biopsy)
Fig5, A g-l	2.81	1.13	1.13 (19)	40.0	44.0	54.5 (19)	Alive (19)
a	2.87	0.96	0.1 (31)	34.1	28.4	36.6 (31)	Alive (31)
b	2.96	2.15	0.71 (19)	56.0	65.0	53.8 (19)	Alive (19)
c	0.31	0	0 (36)	42.9	47.9	52.3 (36)	Alive (36)
d	0.97	0.6	0.46 (10)	41.5	47.4	55.5 (10)	Death of cervical cancer (10)

Supplementary Figure 3-B,C: Expression of HABP in all cases of anti-VEGF therapy-induced glomerular microangiopathy other than a case shown in Figure 5

TKI: tyrosine kinase inhibitor, mAb: monoclonal antibody, VEGFR: vascular endothelial growth factor receptor, VEGFA: vascular endothelial growth factor A

Antibody	Company
Human Hyaluronan Binding Protein, Biotin	Hokudo, Sapporo, Japan
Rabbit anti-Laminin antibody	Abcam, Cambridge, UK
Rabbit anti-synaptopodin antibody	Acris Antibodies GmbH, Herford, Germany
Mouse anti heparan sulfate (10E4 epitope) antibody	United States Biological, Salem, MA
Goat anti-mouse IgG antibody, HRP conjugate	Nichirei Bioscience, Tokyo, Japan
FITC-labeled rabbit anti-mouse IgM	Dako, Santa Clara, CA
FITC-labeled goat anti-mouse IgM	Southern Biotech, Birmingham, AL
Alexa 488-labeled goat anti-rabbit IgG	Thermo Fisher Scientific, Waltham, MA
Alexa 555-labeled goat anti-rabbit IgG	Thermo Fisher Scientific, Waltham, MA
Alexa 555 conjugate streptavidin	Thermo Fisher Scientific, Waltham, MA
Alexa 555-labeled goat anti-mouse IgM	Southern Biotech, Birmingham, AL
DAPI (diamidino-2-phenylindole)	Dojindo, Kumamoto, Japan

Lectin	Company
Tomato fluorescein lycopersicon esculentum lectin	Vector Laboratories, Burlingame, USA

ELISA kit	Company
BCA Protein Assay Kit	Thermo Fisher Scientific, Waltham, MA
Creatinine ELISA assay kit	FUJIFILM Wako Pure Chemical, Osaka, Japan
Mouse Urinary Albumin Assay Kit	FUJIFILM Wako Chemicals, Osaka, Japan
Hyaluronan ELISA assay kit	PG Research, Tokyo, Japan

Supplementary Table 1: List of antibodies and ELISA kits used

	Non-pregnant women of reproductive age	Normal pregnancy group	Pre-eclampsia	***P*-value**
n	8	5	6	
Age, yr	31.6 ± 5.78	36.6 ± 5.46	30.7 ± 5.85	0.218
Daily urine protein, g/day	0.05 ± 0.06	0.08 ± 0.05	6.27 ± 5.68*	0.004
Serum creatinine, mg/dL	0.59 ± 0.07	0.41 ± 0.08*	0.67 ± 0.15	0.002
eGFR, mL/min/1.73m^2	96.4 ± 13.9	142.2 ± 32.0*	88.8 ± 19.3	0.002

Supplementary Table 2: Cases of pre-eclampsia, pregnant woman without pre-eclampsia and healthy woman evaluated for expression of serum levels of hyaluronan

Data are presented as mean ± SD. SD: standard deviation.
* indicates a significant difference compared with non-pregnant women of reproductive age, at a significance level of P< 0.05.
